# Comprehensive Metabolic Profiling of MYC-Amplified Medulloblastoma Tumors Reveals Key Dependencies on Amino Acid, Tricarboxylic Acid and Hexosamine Pathways

**DOI:** 10.3390/cancers14051311

**Published:** 2022-03-03

**Authors:** Khoa Pham, Allison R. Hanaford, Brad A. Poore, Micah J. Maxwell, Heather Sweeney, Akhila Parthasarathy, Jesse Alt, Rana Rais, Barbara S. Slusher, Charles G. Eberhart, Eric H. Raabe

**Affiliations:** 1 Division of Neuropathology, Department of Pathology, School of Medicine, Johns Hopkins University, Baltimore, MD 21205, USA; kpham8@jhmi.edu (K.P.); allison.hanaford@seattlechildrens.org (A.R.H.); brad.a.poore@gmail.com (B.A.P.); ceberha@jhmi.edu (C.G.E.); 2 Division of Pediatric Oncology, Department of Oncology, School of Medicine, Johns Hopkins University, Baltimore, MD 21287, USA; mmaxwel1@jhmi.edu (M.J.M.); hsweene1@jh.edu (H.S.); apartha1@jhu.edu (A.P.); 3 Sidney Kimmel Comprehensive Cancer Center, School of Medicine, Johns Hopkins University, Baltimore, MD 21287, USA; 4 Johns Hopkins Drug Discovery, Baltimore, MD 21205, USA; jalt1@jhmi.edu (J.A.); rrais2@jhmi.edu (R.R.); bslusher@jhmi.edu (B.S.S.); 5 Department of Neurology, School of Medicine, Johns Hopkins University, Baltimore, MD 21205, USA

**Keywords:** Warburg effect, mass spectrometry, isotope labeling, cancer metabolism, pediatric brain tumor

## Abstract

**Simple Summary:**

The oncogene *MYC* alters cellular metabolism. Medulloblastoma is the most common malignant pediatric brain tumor. *MYC*-amplified medulloblastoma has a poor prognosis, and the metabolism of *MYC*-amplified medulloblastoma is poorly understood. We performed comprehensive metabolic profiling of *MYC*-amplified medulloblastoma and found increased reliance on potentially targetable pathways. We also found that the metabolism of *MYC*-amplified cell lines differed from orthotopic brain tumors in vitro and in flank tumors, suggesting that analyses conducted in vitro or in flank tumors may miss key vulnerabilities.

**Abstract:**

Reprograming of cellular metabolism is a hallmark of cancer. Altering metabolism allows cancer cells to overcome unfavorable microenvironment conditions and to proliferate and invade. Medulloblastoma is the most common malignant brain tumor of children. Genomic amplification of *MYC* defines a subset of poor-prognosis medulloblastoma. We performed comprehensive metabolic studies of human *MYC*-amplified medulloblastoma by comparing the metabolic profiles of tumor cells in three different conditions—in vitro, in flank xenografts and in orthotopic xenografts in the cerebellum. Principal component analysis showed that the metabolic profiles of brain and flank high-MYC medulloblastoma tumors clustered closely together and separated away from normal brain and in vitro MYC-amplified cells. Compared to normal brain, *MYC*-amplified medulloblastoma orthotopic xenograft tumors showed upregulation of the TCA cycle as well as the synthesis of nucleotides, hexosamines, amino acids and glutathione. There was significantly higher glucose uptake and usage in orthotopic xenograft tumors compared to flank xenograft tumors and cells in culture. In orthotopic tumors, glucose was the main carbon source for the *de novo* synthesis of glutamate, glutamine and glutathione through the TCA cycle. In vivo, the glutaminase II pathway was the main pathway utilizing glutamine. Glutathione was the most abundant upregulated metabolite in orthotopic tumors compared to normal brain. Glutamine-derived glutathione was synthesized through the glutamine transaminase K (GTK) enzyme in vivo. In conclusion, high *MYC* medulloblastoma cells have different metabolic profiles in vitro compared to in vivo, and key vulnerabilities may be missed by not performing in vivo metabolic analyses.

## 1. Introduction

Malignant transformation is a process that drives normal cells to become cancerous through the accumulation of alterations in proto-oncogenes and tumor suppressors [1,2,3,4]. Among different types of tumors, genetic alterations in PI3/mTOR, RAS/BRAF, MYC and TP53 reprogram metabolic pathways, allowing cancer cells to overcome unfavorable conditions and enabling them to proliferate at a pathologic rate and metastasize [5,6,7,8,9,10,11]. Identifying and interrupting the abnormal metabolic pathways that benefit cancer cells could yield a therapeutic index in which cancer cells are targeted while normal cells are not harmed [12,13,14].

In vitro metabolic studies and flux analysis using stable isotopes can provide a picture of the intracellular metabolite levels and how those metabolites change in response to therapy. However, in vitro models miss the influence from the native microenvironment, such as physiologic or hypoxic oxygen tension and pH and limited availability of nutrients as well as interaction with stromal cells and tumor-associated macrophages, all of which could have a significant impact on the intracellular metabolites of cancer cells [15,16,17,18]. These differences could potentially confound the applicability of cell culture metabolic and therapeutic findings to in vivo tumors, as shown in some type of cancers [19,20,21,22]. We therefore sought to assess how different types of in vitro and in vivo microenvironments affected the metabolic profiles of medulloblastoma.

Medulloblastoma is the most common malignant brain tumor in children. Survival depends on the molecular genetics and epigenetics of the patient’s tumor [23]. *MYC*-amplified medulloblastoma has a worse outcome compared to non-*MYC* amplified medulloblastoma [24]. The poor prognosis for “Group 3”, *MYC*-amplified medulloblastoma patients [25,26] and the severe complications faced by survivors due to the intensity of the therapy they receive indicate an urgent need for more effective and less toxic therapies.

We performed comprehensive metabolic studies—employing liquid chromatography/mass spectrometry (LC/MS) and uniformly labeled glucose and glutamine—of human *MYC*-amplified medulloblastoma by comparing the metabolic profiles of tumor cells in three different environments—in vitro, in flank xenografts and in orthotopic xenografts. Our goals were to: (1) identify changes in the metabolic pathways in the orthotopic tumors compared to normal brain; and (2) test if glucose and glutamine had the same metabolic fates in different tumor cell environments. We hypothesized that due to alterations in oxygen tension, nutrient availability and the microenvironment there would be significant discrepancies between the metabolic profile of the same *MYC*-amplified medulloblastoma cells in vitro, in flank tumors and in orthotopic tumors.

## 2. Materials and Methods

### 2.1. Cell Lines and Culture Conditions

#### 2.1.1. Cell Culture

The patient-derived medulloblastoma cell line D425MED, first established at Duke University, Durham, NC, USA, was grown in MEM media (Gibco, Waltham, MA, USA) supplemented with 5% FBS (Gibco, Waltham, MA, USA) and 1% NEAA (Gibco, Waltham, MA, USA) [27,28,29,30,31]. The MED211 patient-derived xenograft was obtained from the Brain Tumor Resource Lab, Seattle, WA, USA and has been previously described [27,28,29,30]. We developed a cell line from the MED211 PDX model by removing tumor tissue from the tumor as described [28]. MED211 cells were grown in EGF/FGF (Peprotech, Rocky Hill, NJ, USA) neurobasal media.

In vitro metabolic flux experiments involved the media in confluent cells being changed just prior to the experiment. Three biological replicate samples of each cell line were pulsed with 10 μM U-glucose (13C6 99% purity) label from Cambridge Isotope (No. CLM-1396-1) or 4 μM U-glutamine (13C5, 15N2, 99% purity) label from Cambridge Isotope (No. CNLM-1275-H-0.5) for 2 h. Following the pulse, cells were spun down and washed with PBS. 1 mL of 80% UPLC-grade ice cold methanol was added to each pellet. Pellets were vortexed for 1 min and incubated at −80 °C to extract metabolites. Analysis of metabolites is described below.

#### 2.1.2. Animal Studies

Orthotopic xenografting D425MED and MED211 involved the following process. After induction of general anesthesia with ketamine/xylazine in Nu/Nu mice, a burr hole was made in the skull of female Nu/Nu mice Charles River (Wilmington, MA, USA) 1 mm to the right of and 2 mm posterior to the lambdoid suture with an 18 gauge needle. The needle of a Hamilton syringe was inserted to a depth of 2.5 mm into the cerebellum using a needle guard, and 100,000 D425MED cells or MED211 cells in 3 μL of media were injected. MED211 tumors were established by serial transplantation of the patient-derived xenograft and not from cells in culture. All animals were monitored daily until they became symptomatic, exhibiting weight loss, hunching and ataxia. Mice were sacrificed to harvest tumor and uninvolved cerebellum and cortex in the same mouse for histology and metabolic studies.

Prior to tumor implantation, flank xenografting of D425MED and MED211 involved, animals being anesthetized with a mixture of 10% ketamine and 5% xylazine. One million cells of D425MED or MED211 suspended in 200 μL of a 50:50 mix of Matrigel (Corning) and media were injected for each flank tumor. Cells were injected using an 18 gauge needle. One tumor was implanted behind each flank, so each mouse carried four flank tumors [32].

### 2.2. In Vivo Stable Isotope Labeling and Metabolite Extraction and Analyses

Uniformly labeled glutamine was prepared at a 100 μM concentration in PBS and uniformly labeled glucose was prepared as a 20% solution in PBS. Three animals per group were given three 100 μL IP injections of isotope spaced 15 min apart. Euthanasia occurred two hours after the second isotope injection. Tumors were visually identified in the right cerebellar hemisphere due to their more grey/white appearance compared to the normal cerebellum and were dissected and immediately removed and flash frozen in liquid nitrogen. All uniformly labeled isotopes were obtained from Cambridge Isotope Labs, Tewksbury, MA, USA.

Frozen tumors were manually homogenized in liquid nitrogen using a mortar and pestle chilled by dry ice and liquid nitrogen. As the flank tumors were very large, an aliquot of tumor powder was weighed and incubated at −80 °C with 5 volumes of 80% ice-cold HPLC grade methanol to extract metabolites.

### 2.3. Mass Spectrometry Analysis

Samples (both in vivo and in vitro) were centrifuged at 14,000× *g* rpm for 10 min at 4 °C, and the supernatants were transferred to glass insert liquid chromatography vials. Analyses occurred on an Agilent 1290 liquid chromatography system coupled to an Agilent 6520 quadrupole time of flight mass spectrometer. Samples (5 µL) were injected and separated on a Waters Acquity UPLC BEH (bridged ethyl hybrid) Amide 1.7 µm 2.1 × 100 mm HILIC (hydrophilic interaction liquid chromatography) column with a flow rate of 0.3 mL/minute. Mobile phases consisted of A (water + 0.1% formic acid) and B (acetonitrile + 0.1% formic acid). The column was equilibrated at 2.5/97.5 (A/B) and maintained for 1 min post injection. Mobile-phase A increased in a linear gradient from 2.5% to 65% from 1 to 9 min post injection then stepped to 97.5% A from 9 to 11 min to wash the column. Column was equilibrated in starting condition for 3 min before the next injection. The mass spectrometer, equipped with a dual electrospray ionization source, was run in negative ion and then in positive ion mode. The scan range was 50–1600 m/z. The source settings consisted of drying gas flow rate: 11 L/min; nebulizer: 40 pounds per square inch gauge; gas temp: 350 °C; capillary voltage: 3000 V (neg), 2500 V (pos).

#### 2.3.1. Metabolite Analysis

Liquid chromatography–mass spectrometry data were analyzed using Agilent Qualitative Analysis B.07.00 and Elucidata Metabolomic Analysis and Visualization ENgine (El-MAVEN) [33]. Metabolite identification was determined using standards and fragmentation database.

#### 2.3.2. Sample Normalization and Statistical Analysis

Sample normalization and statistical analysis was performed with MetaboAnalyst 5.0 version. Resulting non-labeled and labeled data for each sample were normalized using the total ion count and then logarithmically transformed (base = 10). For each metabolite, transformed values greater than six standard deviations from mean across sample groups were set to missing data. Processing of the raw data yielded 72 identified metabolites. Statistical analysis as well as pathway analysis were performed by the submission of normalized data to a web-based service for metabolic data analysis: MetaboAnalyst (http://www.metaboanalyst.ca/MetaboAnalyst/, accessed in 2021). MetaboAnalyst is a web-based tool that combines results from pathway enrichment analysis with pathway topology analysis, which allowed the identification of the most relevant pathways involved in the conditions under study [34]. Data for identified metabolites detected in all samples were submitted into MetaboAnalyst with annotation based on common chemical names. Verification of accepted metabolites was conducted manually using HMDB, KEGG, and PubChem DBs.

GraphPad Prism was used to represent data graphically and measure statistical significance by Student’s *t*-test.

### 2.4. Human RNAseq Data

RNAseq data were accessed through cBioPortal for Cancer Genomics, with specific queries to the publicly available Pediatric Brain Tumor Atlas, a collaborative effort by Children’s Brain Tumor Tissue Consortium and Pacific Pediatric Neuro-Oncology Consortium with patients and their families. A manuscript describing this dataset is currently in preparation (https://alexslemonade.github.io/OpenPBTA-manuscript/, accessed in January 2022). Raw data were loaded into GraphPad Prism and analyzed by one-way ANOVA with Dunnet’s multiple comparison tests.

### 2.5. Antibodies and Reagents

#### Western Blots

Proteins from cultured cell pellets or snap frozen in vivo samples were homogenized and extracted using RIPA buffer (Millipore Sigma, Burlington, MA, USA) and quantified using a Bradford Assay. We used antibodies against GLUT1 (Novus biologicals (NB110-39113)), glutamine synthetase (Abcam (ab73593)); glutathione synthetase (abcam (ab91591); GTK (KAT1) (Santa Cruz Biotechnologies (sc-374531)), Nit2 (Origene (TA501138)) and beta actin (Santa Cruz (sc-47778)). The following dilutions were used for all primary antibodies (1:1000), beta actin (1:1000). Peroxidase-labeled secondary antibodies were from Cell Signaling Technologies (Danvers, MA, USA) and used at a 1:3500 dilution. Bands were quantified using ImageJ, verson 1.5. Uncropped western blots are included in supplementary material (Figure S5).

## 3. Results

### 3.1. Orthotopic D425MED and MED211 Xenografts Showed Upregulation of Nucleotide Metabolism, Amino Acid, and Glutathione Synthesis

Microscopic examination of hematoxylin and eosin (HE) stained sections confirmed the presence of cellular xenografts, with representative images shown in (Figure S1A,B). Tumors demonstrated a “large cell” histology, commonly associated with MYC amplification. Tumor cells invaded into and disrupted the surrounding cerebellar architecture. These HE stained sections demonstrate that the orthotopic xenograft tumors used in our study grew in the native microenvironment, with histology similar to that of human primary *MYC*-amplified medulloblastoma.

**Figure 1:**
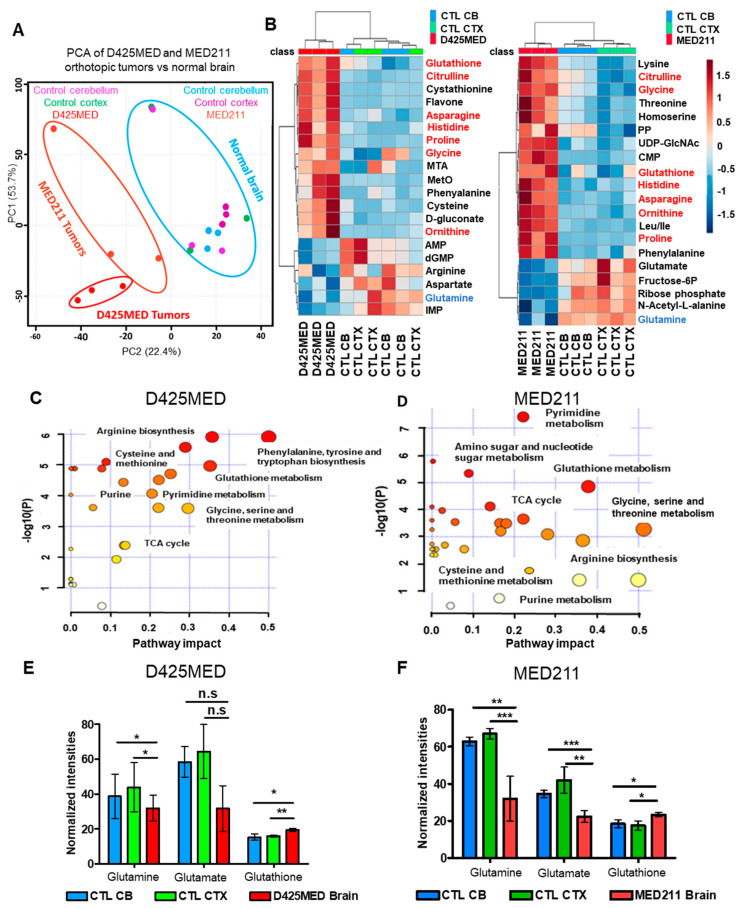
Orthotopic high *MYC*-amplified medulloblastoma tumors show upregulation of TCA cycle activities, nucleotides, amino acids and glutathione synthesis compared to normal brain (**A**). Principal component analysis shows distinct metabolic profiles of high MYC amplified D425MED and MED211 medulloblastoma tumors versus normal brain. D425MED and MED211 tumors segregate from both normal cerebellum and normal cortex. (**B**). Heat map of 20 metabolites that were statistically significantly different in D425MED and MED211 orthotopic tumors compared to normal cortex (CTL CTX) and cerebellum (CTL CB). Commonly increased metabolites in *MYC*-amplified medulloblastoma compared to normal brain included amino acids, glutathione and polyamines such as ornithine. Metabolites upregulated in both D425MED and MED211 compared to normal brain are highlighted in red. Glutamine was decreased in orthotopic tumors compared to normal brain (highlighted in blue). Abbreviations: MTA = S-methyl-5′-thiaoadenosine, MetO = methionine sulfoxide, AMP = adenosine monophosphate, dGMP = deoxyguanine monophosphate, IMP = inosine monophosphate, CMP = cytidine monophosphate, PP = pyrophosphate, UDP-GlcNAc = uridine diphosphate N-acetylglucosamine. (**C**,**D**) Enrichment and pathways analysis using MetaboAnalyst 5.0 showed upregulation of the TCA cycle, glutathione synthesis and the metabolism of arginine, nucleotides and amino acids in both D425MED and MED211 orthotopic tumors. The y-axis is the log10 *p* value and the x-axis represents the pathway impact value computed from pathway topological analysis. The color and the size of the circles are based on the *p*-value and the number of hits. Larger circles indicate more metabolites are upregulated in that pathway and more red color indicates increasing statistical significance. (**E**,**F**) The three most abundant metabolites found in orthotopic D425MED (**E**) and MED211 (**F**) tumors compared to normal brain were glutamine, glutamate and glutathione. Glutamine and glutamate were observed at lower levels compared to normal brain, but the ratio of glutamate/glutamine was significantly higher in tumor compared to normal brain, suggesting higher glutamine usage in tumors. Glutathione (GSH) was also upregulated in tumors compared to normal brain. Abbreviations: normal (control) cerebellum = CTL CB; normal (control) cortex = CTL CTX; D425MED orthotopic tumor = D425MED; MED211 orthotopic tumor = MED211. The bar graphs show the mean intensities with the SD as error bar. Each group has three biological replicate samples. * *p* < 0.05, ** *p* < 0.01, *** *p* < 0.001, Student’s *t*-test, n.s. = not significant.

Metabolic analysis found 3000–4000 analytes in normal brain (normal cortex and contralateral and uninvolved cerebellum) and orthotopic *MYC*-amplified D425MED and MED211 medulloblastoma tumors. Principal component analysis of metabolomes found distinct metabolic profiles in normal brain (cortex and normal contralateral cerebellum) compared to *MYC*-amplified medulloblastoma D425MED and MED211 orthotopic tumors (Figure 1A). In one of the orthotopic MED211 tumors, we found that the tumor and normal brain metabolomes data were distinct from the other samples in the PCA, likely due to technical issues. However, the PCA demonstrated that even with this heterogeneity, the normal samples clustered together with clear separation from the MED211 tumor samples. We also found that the D425MED and MED211 orthotopic tumor samples clustered together and were distinct from all of the normal brain samples. Analysis of the known or targeted metabolites further showed clear separation between normal brain and the orthotopic tumors. There were 72 metabolites confirmed with fragmentation data based in both normal brain and tumors (52 upregulated and 20 downregulated metabolites compared to normal brain). A heat map of the top 20 statistically significantly different metabolites showed upregulation of glutathione, ornithine, citrulline, histidine, proline, glycine and asparagine in orthotopic tumors compared to normal cortex and cerebellum (Figure 1B). Interestingly, orthotopic medulloblastoma tumors had lower glutamine levels compared to normal brain.

The enrichment and pathway impact analysis of the 52 upregulated metabolites revealed that the activity of the TCA cycle as well as the synthesis of nucleotides, glutathione and amino acids were upregulated in tumors compared to normal brain (Figure 1C,D). Of novel therapeutic interest, we also identified multiple metabolites of the urea cycle as being upregulated in medulloblastoma orthotopic tumors compared to normal brain, and this manifested as showing alteration in arginine metabolism in C and D. Glutamate, glutamine and glutathione were the most abundant metabolites detected in both tumors and normal brain. However, glutamate and glutamine were lower in tumors when compared to normal brain whereas glutathione was the most abundant upregulated metabolite found in tumors (Figure 1E,F).

### 3.2. The Metabolic Profile of MYC-Amplified Medulloblastoma In Vitro Models Was Distinct from In Vivo Flank and Orthotopic Xenograft Tumor Models

**Figure 2:**
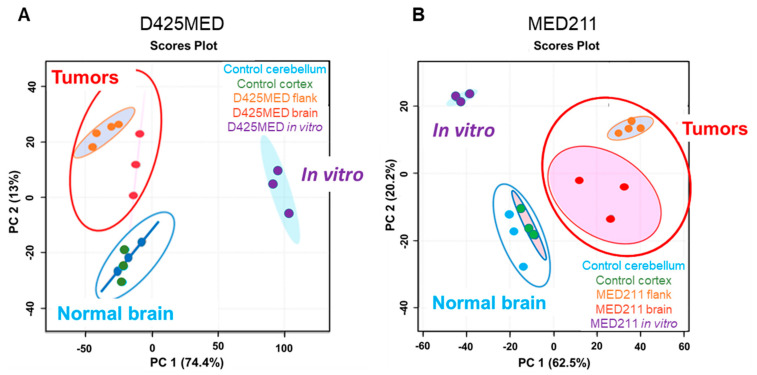
(**A**,**B**). Principal component analysis shows the metabolomes of orthotopic and flank xenograft tumors of D425MED (**A**) and MED211 (**B**) cluster closely together and are separated from normal brain and the metabolome of D425MED and MED211 cells grown in an in vitro environment. Abbreviations: normal (control) cerebellum = CTL CB; normal (control) cortex = CTL CTX; D425MED orthotopic tumor = D425MED brain; D425MED flank xenograft tumor = D425MED flank; MED211 orthotopic tumor = MED211 brain; MED211 flank xenograft tumor = MED211 flank.

A major aim of our study was to learn how cancer cells alter their metabolic pathways to adapt to different growth environments, and the degree to which in vitro models recapitulated in vivo conditions. We therefore performed PCA comparing known metabolites of D425MED and MED211 MYC—amplified medulloblastoma in three different environments: in vitro, flank xenograft and orthotopic xenograft tumors growing in the cerebellum. The analysis showed that the metabolic profiles of in vivo settings (orthotopic and flank xenograft tumors) clustered closely together, but separated away from normal brain and the profile of the in vitro models (Figure 2A,B). We applied stable isotope uniformly labeled glucose (13C6) and glutamine (13C515N2) metabolomics to further understand how cancer cells utilize these substrates in different tumor environments.

### 3.3. Glucose Is the Main Carbon Source Fueling the TCA Cycle in Normal Brain and Orthotopic D425MED High MYC Medulloblastoma

**Figure 3:**
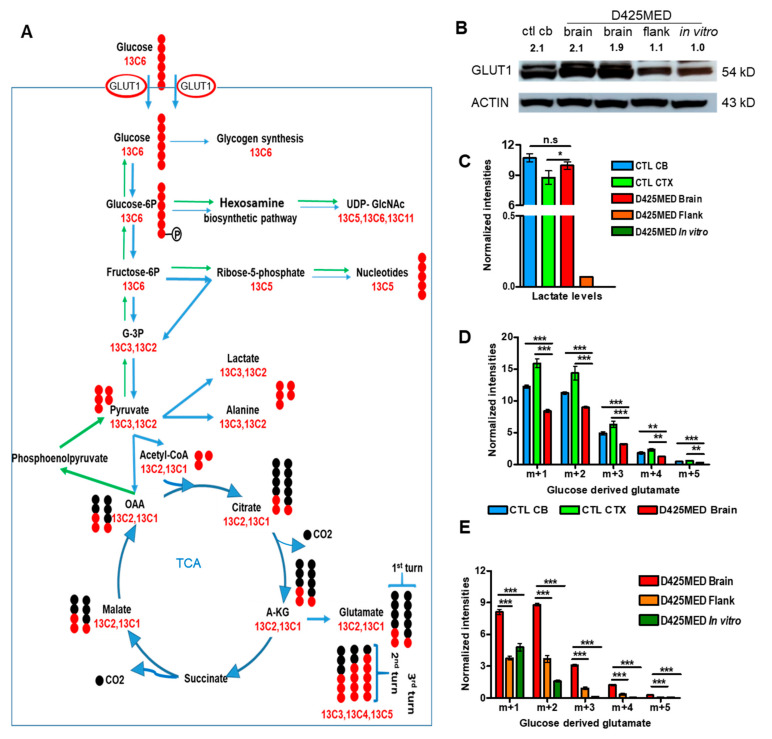
Glucose is the main carbon source for the TCA cycle in MYC-amplified medulloblastoma in vivo and xenograft tumors actively synthesize glutamate from glucose. (**A**) Cartoon illustrating the tracing method of uniformly labeled 13C6 glucose after being transported into cells by the glucose transporter GLUT1 and its contribution to different metabolic pathways. Red dots represent stable isotope labeled carbon C13 with one extra mass on the regular carbon 12C. Black dots represent for unlabeled carbon, or 12C. By tracing down the number of labeled carbon 13C (from uniformly labeled glucose 13C6) appearing in the downstream glucose-derived metabolites, we are able to tell how much glucose carbon contributes to the TCA cycle, glutamate synthesis, and other metabolites through different metabolic pathways. (**B**) Western blot showing higher expression of the glucose transporter GLUT1 (encoded by SLC2A1) in normal brain (ctl cb) and orthotopic tumor (D425MED brain) versus flank tumor (D425MED flank) and in vitro D425MED cells. Numbers above the blot indicate the densitometry normalized to ACTIN and compared to the in vitro condition. Uncropped western blots are included in supplementary materials. (**C**) Bar graph showing increased lactate intensities in normal brain (NCB and CTX) and D425MED orthotopic tumors compared to flank tumors and D425MED cells in culture (in vitro). (**D**) Bar graph showing glucose-derived glutamate levels in normal brain (NCB and CTX) and lower levels in D425MED Brain (orthotopic tumor). Glutamate was the most abundant glucose-derived metabolite found in tumor and normal brain. (**E**) Bar graph showing increased glucose-derived glutamate levels in orthotopic tumor (D425MED Brain) compared to flank tumor and D425MED cells in culture. The highest intensities of glucose derived glutamate were found in orthotopic xenograft tumors (m + 1, m + 2, m + 3) compared to flank tumors and D425MED cells in culture. Abbreviations: normal (control) cerebellum = CTL CB; normal (control) cortex = CTL CTX; D425MED orthotopic tumor = D425MED brain; D425MED flank xenograft tumor = D425MED flank; D425MED cells in culture = D425MED in vitro. TCA = tricarboxylic acid. The bar graph shows the mean intensities with the SD as error bar. Each group has three biological replicate samples. * *p <* 0.05, ** *p* < 0.01, *** *p* < 0.001, Student’s *t*-test.

Glucose is the main energy source in the normal brain. After entering cells, glucose is converted to glucose-6-phosphate, which is used in different metabolic pathways, such as the pentose phosphate pathway (PPP), the hexosamine biosynthetic pathway (HBP), amino acid synthesis, glycolysis and oxidative phosphorylation (Figure 3A). In normal brain, glucose is one of the critical energy sources, and normal brain expresses high levels of the glucose transporter GLUT1 (encoded by *SLC2A1)*. We detected increased levels of GLUT1 in normal brain and in orthotopic medulloblastoma tumor compared to flank tumors (Figure 3B).

While glucose is a key energy source in normal brain, the normal brain also produces lactate at high levels. Lactate is one of the products of glycolysis that is highly present in normal brain and is transported from glia to neurons to provide an additional energy source through the astrocyte–neuron lactate shuttle [35]. We detected much higher lactate in normal brain and in orthotopic medulloblastoma tumor than in flank tumors or cells in culture (Figure 3C).

We then applied uniformly labeled glucose to study the contribution of glucose carbons into different downstream metabolic pathways. Isotope tracing using uniformly labeled glucose (13C6, m + 6) showed that glucose carbons contributed significantly to glutamate synthesis (which had the highest intensities of labeled 13C among other downstream glucose derived metabolites) in orthotopic xenograft tumor and normal brain (Figure 3D). The glucose-derived pyruvate m + 2 (derived through the pentose phosphate pathway) and m + 3 (generated by glycolysis) lose one carbon to yield Acetyl-coA m + 1 and m + 2 when entering the TCA cycle. As a result, cells yield m + 1 and m + 2 glucose-derived glutamate, as the product of the first completed TCA cycle turn. There were also glucose-derived glutamate m + 3, m + 4 and m + 5 (which are derived from citrate m + 3, m + 4, m + 5 as the products of m + 1, m + 2, m + 3 OAA combining with Acetyl-coA m + 1, m + 2) in normal brain and MYC-amplified medulloblastoma tumors, that represented the products of the TCA after the second turn and third turns. These data demonstrate that glucose is incorporated into the TCA cycle in high MYC medulloblastoma orthotopic tumors. There was even higher glucose incorporation into the TCA cycle in orthotopic tumors compared to normal brain, and this was confirmed upon analysis of the TCA cycle intermediate metabolites found in tumor and normal brain (Figure S2A). These findings confirmed that tumor cells simultaneously use glycolysis and oxidative phosphorylation.

We found the highest intensities of glucose-derived glutamate in orthotopic xenograft tumors, indicating that these tumors robustly synthesize glutamate through the TCA cycle. By comparing the intensities of glutamate isotopologues and other intermediate metabolites found in the TCA cycle in our three different settings, we found there was significantly higher glucose anaplerosis in orthotopic tumors compared to flank xenograft tumors and in vitro culture (Figure 3E, Figure S2B). These findings show that orthotopic D425MED *MYC*-amplified medulloblastoma and the control normal brain had higher uptake and use of glucose compared to D425MED flank xenograft tumors or D425MED cells in culture.

### 3.4. Glucose-Derived Glutamate Was Used Differently in the In Vitro vs. In Vivo Setting

**Figure 4:**
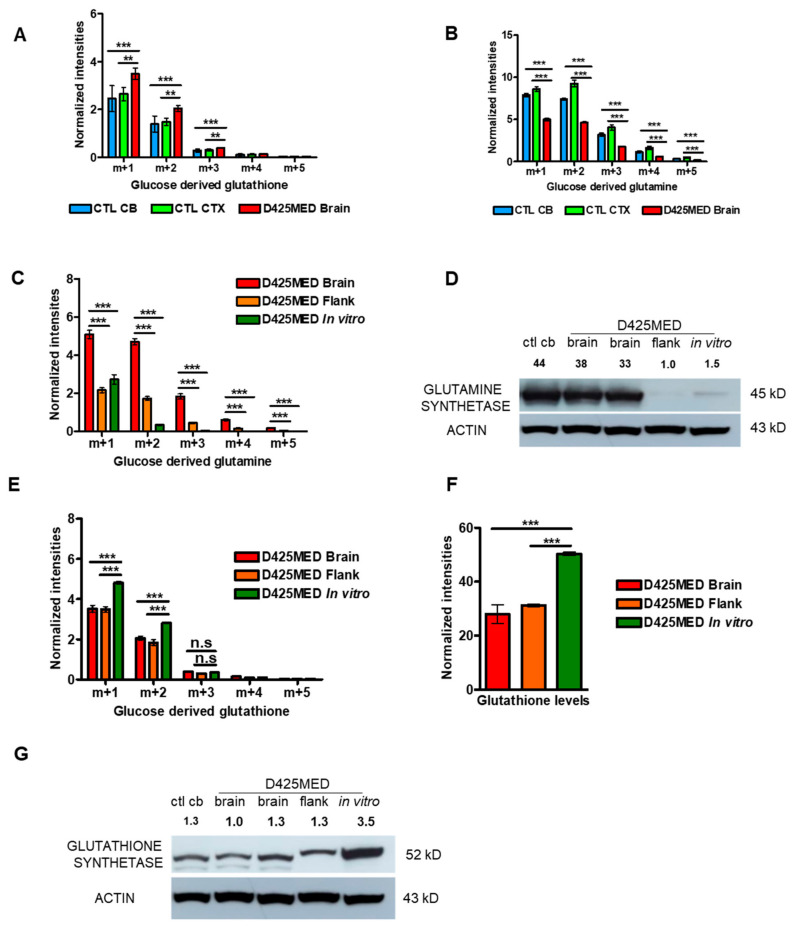
Glucose is the main carbon source for the TCA cycle in *MYC*-amplified medulloblastoma in vivo and xenograft tumors actively synthesize glutamine and glutathione. (**A**) Bar graph showing increased glucose-derived glutathione in orthotopic tumor compared to normal brain. (**B**) Bar graph showing lower intensities of glucose-derived glutamine found in orthotopic tumor compared to normal brain. (**C**) Bar graph showing increased m + 1, m + 2, m + 3 glucose-derived-glutamine in orthotopic D425MED tumors compared to flank tumors and cells in culture. (**D**) Western blot showing increased glutamine synthetase expression in normal cerebellum (ctl cb) and orthotopic tumor (D425MED brain) compared to flank tumor and D425MED cells in in vitro. Numbers above the graph indicate densitometry of the band normalized to ACTIN, compared to the “flank” condition. (**E**) Bar graph showing increased m + 1, m + 2, m + 3 glucose-derived glutathione in D425MED cells in vitro compared to orthotopic and flank tumors. (**F**) Bar graph showing unlabeled glutathione levels are highest in D425MED cells in culture compared to flank and orthotopic tumors. (**G**) Western blot showing increased glutathione synthetase in D425MED cells in culture compared to flank and orthotopic tumors and normal brain (ctl cb). Numbers above the blot indicate densitometry normalized to ACTIN and compared to the lowest expression in normal brain. Uncropped western blots are included in supplementary materials. Abbreviations: normal (control) cerebellum = CTL CB; normal (control) cortex = CTL CTX; D425MED orthotopic tumor = D425MED brain; D425MED flank xenograft tumor = D425MED flank. The bar graph shows the mean intensities with the SD as error bar. Each group has three biological replicate samples. n.s. not significant, ** *p* < 0.01, *** *p* < 0.001, Student’s *t*-test.

Glutamate is a major excitatory neurotransmitter in the brain [36]. Glutamate can also be converted to glutamine by glutamine synthetase (GS) [37,38,39] or incorporated into glutathione synthesis [40]. We detected significantly higher glucose-derived glutathione in tumors compared to normal brain, indicating that *MYC*-amplified medulloblastoma was using carbons from glucose to synthesize glutamate, which was then being incorporated into glutathione and glutamine (Figure 4A,B). Interestingly, we observed decreased m + 1, m + 2, and m + 3 glucose-derived glutamate and glutamine in orthotopic tumor compared to normal brain, but increased m + 1, m + 2, and m + 3 glutathione. One explanation for this would be that the tumor cells have increased glutathione needs and so are preferentially shunting the glucose carbons into glutathione, rather than allowing them to accumulate in glutamate and glutamine.

We also found significantly higher glutamine synthesis in orthotopic tumors compared to flank tumors and cells in culture. We found increased glucose-derived glutamine isotopologue intensities as well as increased glutamine synthetase (GS) protein expression by Western blot in orthotopic brain tumor compared to flank or cells in culture (Figure 4C,D). Although orthotopic tumors used glucose carbons to synthesize glutathione to a greater degree than normal brain, in comparing orthotopic, flank and in vitro D425MED, we found that in vitro tumor cells incorporated glucose carbons to the greatest extent into glutathione (Figure 4E). We confirmed that in vitro D425MED had the highest glutathione levels with non-labeled glutathione intensities (Figure 4F). Western blot showed increased expression of glutathione synthetase in cells in culture compared to orthotopic or flank D425MED tumor and normal cerebellum (Figure 4G), which was consistent with an increased glutathione production in cells in culture compared to orthotopic or flank tumors.

### 3.5. Gluconeogenesis Contributed to the Hexosamine Biosynthetic Pathway and Was Upregulated in Orthotopic D425MED High-MYC Medulloblastoma Tumor Compared to Normal Brain

**Figure 5:**
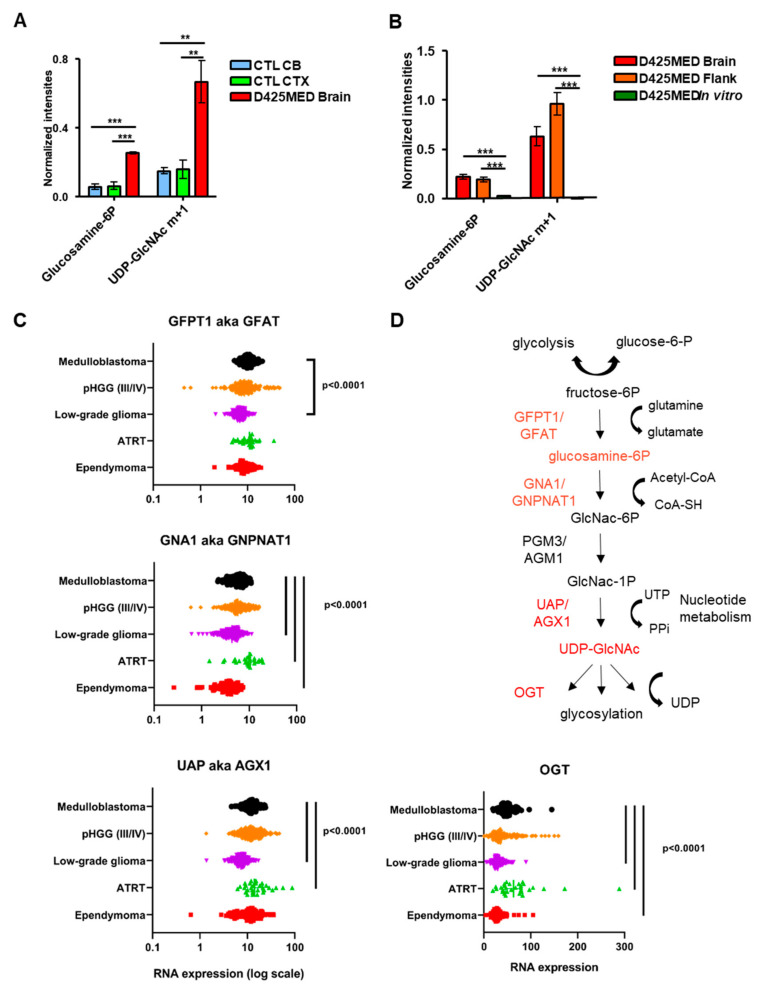
Upregulation of hexosamine biosynthetic pathway in the D425MED orthotopic tumor. (**A**) Bar graph showing that intermediate metabolites found in the hexosamine pathway were significantly higher in tumor compared to normal brain. Total levels of glucosamine-6-phosphate were increased in the D425MED tumor compared to normal brain. We also detected increased levels of Uridine diphosphate-N-acetyl Glucosamine (UDP-GlcNAc) in our studies of incorporation of uniformly labeled glucose. Metabolites were found mostly as m + 1 isotopes, suggesting they were generated through gluconeogenesis. (**B**) Bar graph showing increased hexosamine pathway metabolites in flank and orthotopic D425MED compared to cells in culture. We found increased Glucoseamine-6P and m + 1 UDP-GlcNAc in orthotopic and flank tumors compared to D425MED in culture. The bar graph shows the mean intensities with the SD as error bar. Each group has three biological replicate samples. ** *p* < 0.01, *** *p* < 0.001, Student’s *t*-test. (**C**) Interrogation of the Children’s Brain Tumor Network Pediatric Brain Tumor Atlas RNAseq dataset showed that primary medulloblastoma tumors have increased expression of enzymes in the hexosamine biosynthetic pathway, including *GFAT, GNA1, UAP* and *OGT,* compared to other pediatric brain tumors, particularly low-grade glioma. Bars indicate statistical significance by one-way ANOVA with Dunnet multiple comparisons correction. ATRT tumors had increased average expression of *GNA1*, *UAP,* and *OGT* compared to medulloblastoma. (**D**) Cartoon showing the hexosamine biosynthetic pathway, with metabolites and their corresponding enzymes that are upregulated in medulloblastoma tumors highlighted in red. The bi-directional arrow at top indicates that glycolysis is reversible and gluconeogenesis may also occur. Abbreviations: Normal (control) cerebellum = CTL CB; Normal (control) cortex = CTL CTX; D425MED orthotopic tumor = D425MED brain; D425MED flank xenograft tumor = D425MED flank. ATRT = atypical teratoid/rhabdoid tumor; pHGG = pediatric high-grade glioma.

Orthotopic *MYC*-amplified medulloblastoma D425MED tumors had increased levels of glucosamine-6-phosphate compared to normal brain, suggesting increased reliance on the hexosamine biosynthetic pathway, which is the main pathway used for glycosylation of proteins [41] (Figure 5A). Consistent with increased activity of the hexosamine pathway, we detected increased incorporation of glucose carbons in uridine diphosphate n-acetylglucosamine (UDP-GlcNAc) in our in vivo orthotopic xenografts compared to normal brain (Figure 5A). These metabolites were generated through the gluconeogenesis pathway because m + 1 was the most abundant intensity found among the isotopologues. The HBP is more active in vivo compared to in vitro, with significantly increased total Glucosamine-6P and m + 1 UDP-GlcNAc in flank and orthotopic xenografts compared to cells in culture (Figure 5B).

Interrogation of the Children’s Brain Tumor Network/KidsFirst Pediatric Brain Tumor Atlas RNAseq data showed increased expression of mRNAs encoding key enzymes of the hexosamine biosynthetic pathway in medulloblastoma compared to other pediatric brain tumors (Figure 5C). Specifically, compared to pediatric low-grade glioma, we detected in medulloblastoma increased *GFPT1*, which encodes for the enzyme GFAT that converts fructose-6P to glucosamine-6P, the rate-limiting step in hexosamine synthesis [41]. We also found upregulated *GNA1*, which encodes enzyme that converts glucosamine-6P to N-acetylglucosamine 6P (GlcNac-6P). We found increased RNA levels of *AGX1,* which encodes the enzyme that converts GlcNac-1P to UDP-GlcNAc, as well as *OGT*, an enzyme that transfers N-acetyl-glucosamine (GlcNAc) to proteins. Figure 5D shows a cartoon overview of the pathway highlighting the metabolites we identified as upregulated and the corresponding upregulated enzymes.

### 3.6. The Glutaminase II Pathway Was the Main Pathway Metabolizing Glutamine In Vivo

Glutamine is another major nutrient source for cells. Glutamine is used to synthesize amino acids, nucleotides and glutathione [42]. After conversion to alpha-ketoglutarate, glutamine can replenish the TCA cycle. We used uniformly labeled glutamine (13C5, 15N2) to understand how high MYC medulloblastoma in different environments metabolized glutamine.

**Figure 6:**
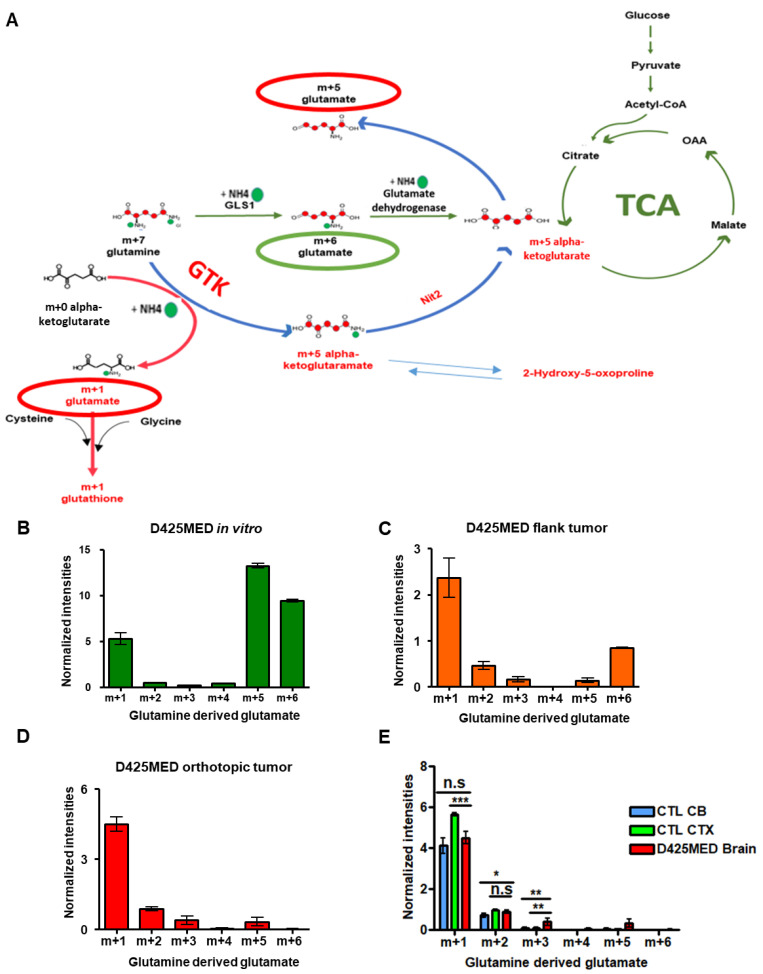
D425MED orthotopic tumors preferentially use the glutaminase II (GTK) pathway over the glutaminase 1 (GLS1) pathway. (**A**) Cartoon illustrating how glutamine is metabolized through GLS1 to yield glutamate m + 6 isotope (green arrows). The glutaminase II pathway (blue arrows) uses glutamine transaminase K (GTK) to generate glutamate m + 1 by adding the amino group from labeled glutamine to alpha-KG. The Nit2 enzyme converts m + 6 alpha-ketoglutaramate (KGM) to m + 5 alpha-KG. This can in turn be converted to m + 5 glutamate by glutamate dehydrogenasae. (**B**) Bar graph showing glutamine-derived glutamate in D425MED cells in vitro, with predominance of m + 6. (**C**) Bar graph showing glutamine-derived glutamate in D425MED cells in flank tumors, showing increasing prominence of m + 1. (**D**) Bar graph showing glutamine-derived glutamate in D425MED cells in orthotopic tumors, showing predominance of m + 1 and near-absence of m + 6. (**E**) Bar graph comparing glutamine-derived glutamate in D425MED cells in orthotopic tumors and normal brain, showing predominance of m + 1 isotopologue. Abbreviations: normal (control) cerebellum = CTL CB; normal (control) cortex = CTL CTX; D425MED orthotopic tumor = D425MED brain; D425MED flank xenograft tumor = D425MED flank. The bar graph shows the mean intensities with the SD as error bar. Each group has three biological replicate samples. n.s. not significant, * *p <* 0.05, ** *p* < 0.01, *** *p* < 0.001, Student’s *t*-test.

The glutaminase 1 pathway is the most known pathway of glutamine metabolism. In this pathway, the glutaminase 1 (GLS1) enzyme converts glutamine to glutamate [43,44]. However, there is another series of enzymatic reactions (starting with glutamine transaminase K (GTK also known as KYAT1) followed by NIT2 called the “glutaminase II pathway” that is the main pathway to utilize glutamine in the brain [32,45,46,47]. In Figure 6A, we show how uniformly labeled glutamine (m + 7) is utilized under both pathways to generate glutamate isotopologues.

We found glutamine-derived glutamate was metabolized through both glutaminase 1 (yielding m + 6) and glutaminase ii pathways (yielding m + 1, m + 5) in cultured D425MED cells (Figure 6B). However, D425MED flank xenograft tumors showed a different pattern of glutamate isotopologues (Figure 6C) in which glutamate m + 1 was the most abundant. In the orthotopic D425MED tumor, this pattern became even more biased toward the glutaminase II pathway, in that almost all of the glutamine-derived glutamate was m + 1 and m + 5, and there was virtually undetectable amounts of glutamate m + 6. The glutaminase II pathway was also predominant in normal cortex and cerebellum (Figure 6D,E). The changing pattern among glutamine-derived glutamate isotopologues showed that most glutamine-derived glutamate was generated through the glutaminase II pathways (GTK) in vivo. We found similar changes in MED211 high MYC amplified medulloblastoma in flank xenograft tumors compared to MED211 cells in culture (Supplemental Figure S3A,B).

In the brain and orthotopic MYC-driven medulloblastoma tumor in addition to m + 1, we also detected at a much lower level in the orthotopic tumor, m + 2, m + 3 and m + 5 glutamate. These other species may represent glutamine carbons that were incorporated into the TCA cycle through alpha-ketoglutamate and then cycled back out after several turns to resynthesize glutamine. Alternatively, the m + 2 and m + 3 isotopologues may represent glutamine carbons that were incorporated in another organ in the mouse into glucose through gluconeogenesis. The resulting circulating glucose then contributed to glutamate and glutamine in tumor and brain via glycolysis and the TCA cycle. However, other glutamine-derived TCA metabolites were virtually undetectable in vivo.

### 3.7. Glutamine Derived Glutathione Synthesis Was Mainly through GTK, and It Was Upregulated in Orthotopic D425MED High MYC Xenograft Tumors Compared to Normal Brain

**Figure 7:**
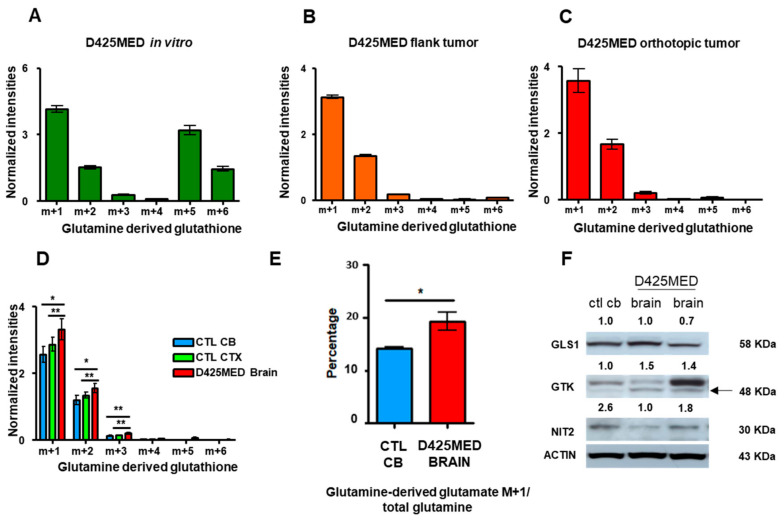
GTK is the main enzyme responsible for glutathione synthesis in vivo. (**A**–**C**). Bar graphs showing the contribution of glutamine-derived carbons and nitrogens to glutathione in D425MED in different environments. The majority of glutathione was synthesized through GTK because the highest glutamine-derived glutathione was the m + 1 isotopologue, in all models. The predominance is most stark in orthotopic tumors, where the m + 6 isotopologue is virtually undetectable. (**D**) Bar graph showing increased glutamine-derived glutathione in orthotopic tumor compared to normal brain. (**E**) Bar graph showing m + 1 glutamate to glutamine ratio in D425MED cells in orthotopic tumors. (**F**) Western blot of glutaminase II pathway enzymes showing increased GTK (arrow) in D425MED orthotopic tumors (D425MED brain) compared to uninvolved cerebellum (CTL CB). The upper band is likely non-specific. ACTIN shows equal loading in all lanes. In contrast to GTK, we did not detect increased NIT2 or GLS1 protein expression MED tumor compared to normal cerebellum. Numbers above each blot show densitometry normalized to ACTIN. Abbreviations: normal (control) cerebellum = CTL CB; normal (control) cortex = CTL CTX; D425MED orthotopic tumor = D425MED brain; D425MED flank xenograft tumor = D425MED flank. The bar graphs show the mean intensities with the SD as error bar. Each group has three biological replicate samples. * *p* < 0.05, ** *p* < 0.01, Student’s *t*-test.

For in vitro and in vivo D425MED, the most abundant labeled glutathione isotopologue was m + 1 (Figure 7A–C). Glutamate m + 1 derived glutathione was synthesized mainly through the glutaminase II pathway. The glutamine transaminase K (GTK or KYAT1) enzyme used endogenous alpha ketoglutarate (alpha-keto acid of glutamate) to incorporate the amino group from uniformly labeled glutamine to form glutamate m + 1. This m + 1 glutamate, together with cysteine and glycine, formed glutathione m + 1 (as demonstrated in the cartoon in Figure 6A). We found similar changes in MED211 in cell culture and flank xenograft tumors (Figure S3C,D).

Orthotopic tumors had significantly higher m + 1 glutamine-derived glutathione compared to normal brain (Figure 7D). Increased activity of GTK in orthotopic tumors was also confirmed with the ratio of m + 1 glutamate to total glutamine in D425MED tumor compared to normal cerebellum (Figure 7E). Western blot of GLS1, GTK, and NIT2 showed increased expression of GTK in orthotopic D452MED tumors compared to normal brain. (Figure 7F).

To extend our findings to additional medulloblastoma tumors, we interrogated the Children’s Brain Tumor Network/KidsFirst Pediatric Brain Tumor Atlas RNAseq dataset (PedscBioportal). We found that medulloblastoma expresses significantly higher mRNA for *KYAT*, the gene that encodes GTK compared to other pediatric brain tumors, including ependymoma, low-grade glioma and atypical teratoid/rhabdoid tumor (ATRT). Expression of *KYAT* was not statistically significant compared to pediatric high-grade glioma (Figure S4).

### 3.8. Overall Model of the Metabolomics of Orthotopic MYC-Amplified Medulloblastoma Reveals Key Dependencies That May Be Therapeutically Targetable

**Figure 8:**
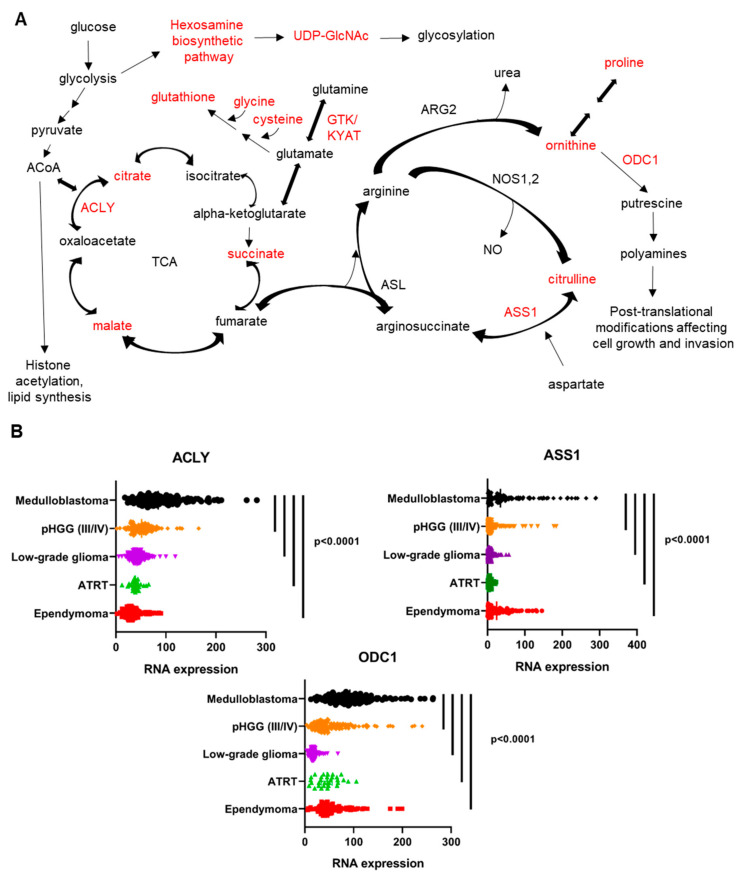
A Cartoon overview of some of the metabolic vulnerabilities identified in our study. Metabolites that we identified as being upregulated in orthotopic MYC-amplified medulloblastoma compared to normal brain are highlighted in red. We identified upregulation of the hexosamine biosynthetic pathway, which is key for the glycosylation of proteins. We also found increased glutathione and elevated levels of the amino acids glycine and cysteine that are combined with glutamate to produce glutathione. We identified upregulation of TCA cycle intermediates succinate and malate and detected glucose-derived carbons in these metabolites, indicating that oxidative phosphorylation was likely occurring in orthotopic tumors. The glutaminase II pathway, featuring glutamine transaminase K (GTK) (encoded by the gene *KYAT)* is the predominate glutaminase pathway in the brain and in orthotopic MYC-amplified tumors. The enzyme ATP citrate lyase (ACLY) converts citate to oxaloacetate and acetyl-CoA, fueling fatty acid biosynthesis and also facilitating the reversal of the TCA cycle. The cartoon highlights the interaction between the tricarboxylic acid (TCA) cycle and urea cycle, in which fumarate shuttles between the two metabolic pathways through the reversible activity of arginosuccinate lyase (ASL). We identified upregulation of the urea cycle intermediates citrulline and ornithine. Citrulline is generated by the degradation of arginine during nitric oxide (NO) production. The enzyme arginosuccinate synthetase 1 (ASS1) combines citrulline with aspartate to generate arginosuccinate. Arginase 2 (ARG2) converts arginine to ornithine, releasing urea. Ornithine can also be synthesized in several steps from proline, which was also increased in *MYC*-amplified medulloblastoma compared to normal brain. Ornithine is a precursor of polyamines, which are intermediates for post-translational protein modification. Ornithine decarboxylase (ODC1) converts ornithine to putrescine, the first step in polyamine synthesis. Outside of the liver, the urea cycle does not generate citrulline from ornithine due to the low expression of ornithine transcarbamylase. Analysis of the Children’s Brain Tumor Network Pediatric Brain Tumor Atlas RNAseq dataset shows increased expression of the mRNA encoding ATP citrate lyase (ACLY), arginosuccinate synthetase 1 (ASS1), and ornithine decarboxylase (ODC1) in medulloblastoma compared to other pediatric brain tumor subtypes. (**B**). *p* values indicated at the right represent results of one-way ANOVA with Dunnet multiple comparisons correction. Each dot represents RNA from a single tumor sample. The vertical bar in each tumor type shows the mean intensity of normalized RNA expression. Metabolic enzymes that we identified as being upregulated in 8B or in other figures in this paper are highlighted in red in (**A**).

Our metabolic analysis of MYC-amplified medulloblastoma revealed upregulation in the metabolism of nucleotides, glutathione, the hexosamine biosynthetic pathway, the urea cycle and amino acids compared to normal brain. We also found increases in TCA cycle components malate, succinate and fumarate. Figure 8A shows a cartoon of the relationship between the TCA and urea cycles, with metabolites found to be upregulated in orthotopic MYC-amplified tumors highlighted in red. Outside of the liver, the urea cycle is not complete, in that there is no expression of the enzyme ornithine transcarbamylase (OTC1) that converts ornithine to citrulline and scavenges ammonia [48,49,50]. The urea cycle is a critical synthetic pathway to produce polyamines by production of ornithine. The urea cycle also produces the signaling molecule nitric oxide (NO) from arginine [48], generating citrulline. Citrulline is combined with aspartate by arginosuccinate synthetase (ASS1) to produce arginoosuccinate. The TCA cycle and urea cycle are linked by fumarate, which shuttles between the pathways in a reversible fashion catalyzed by the enzyme arginosuccinate lyase (ASL) [51]. ASL combines arginosuccinate and fumarate to make arginine.

While we did not detect increased arginine itself, we did identify increased citrulline and ornithine in MYC-amplified orthotopic tumors compared to normal brain. Ornithine is produced by arginase (ARG) or separately in several enzymatic steps from proline. ARG2 is the arginase enzyme most highly expressed in brain tissue [52]. Proline was also increased in MYC-amplified medulloblastoma compared to normal brain. Ornithine is subsequently incorporated into the polyamine biosynthetic pathway by ornithine decarboxylase (ODC1). Polyamines are post-translational protein modifications that promote invasion and growth of cancer cells [53].

To determine if some of the metabolic pathways we identified might be active in human patients with medulloblastoma, we queried the Children’s Brain Tumor Network/KidsFirst Pediatric Brain Tumor Atlas RNAseq dataset (PedscBioportal). We identified increased mRNA levels of *ACLY*, *ASS1* and *ODC1* in medulloblastoma primary tumors compared to other pediatric brain tumors, suggesting an increased reliance in medulloblastoma on polyamines, arginine biosynthesis and the TCA cycle/production of acetyl-CoA (Figure 8B). Of note, we did not detect increased expression in medulloblastoma compared to other pediatric brain tumors of mRNA for pyruvate carboxylase (which would allow direct incorporation of pyruvate carbons to oxaloacetate) or any of the other TCA cycle or urea cycle enzymes).

## 4. Discussion

We identified significant differences in glucose and glutamine metabolism in high MYC medulloblastoma comparing in vitro, flank xenografts and orthotopic xenograft medulloblastoma models. *MYC*-amplified D425MED and MED211 medulloblastoma orthotopic xenograft brain tumors upregulated nucleotide, hexosamine, amino acid and glutathione synthesis compared to normal brain. Glutathione was the most abundant upregulated metabolite found in tumors compared to normal brain. Our findings were consistent with recently reported proteomics data in *MYC*-amplified medulloblastoma, showing upregulation of the glutathione biosynthetic pathway compared to non-MYC-amplified medulloblastoma and normal brain [54].

We found significantly higher glucose uptake and usage in normal brain and orthotopic xenografts compared to flank xenografts and in cells in culture. Glycolysis and incorporation of glucose into the TCA cycle were concurrently found in all settings. Similar findings showing incorporation of glucose carbons into the TCA cycle were reported in metabolic profiling of orthotopic glioblastomas [55].

Medulloblastoma tumors in our studies had higher activity of glucose anaplerosis compared to normal brain. D425MED orthotopic xenografts exhibited the strongest oxidative phosphorylation activities, even at presumably the lowest oxygen tension (3–5% in the brain compared to 21% for cells in culture) [56]. Glucose was the main carbon source for glutamate synthesis through the TCA cycle in high MYC amplified medulloblastoma. This finding confirms robust metabolism of glucose through glycolysis and into the TCA cycle in cancer cells in orthotopic xenografts. The incorporation of glucose into the TCA cycle contradicts a key claim of the “Warburg effect”, namely that cancer cells are reliant on glycolysis for ATP generation and that glucose carbons would not be significantly incorporated into the TCA cycle [57,58].

Rather than being reliant on glutamine from the microenvironment, we found that MYC-amplified medulloblastoma tumor cells synthesized glutamine. Keeping with this theme of glutamine synthesis, the majority of glutamate in vivo became the precursor for glutamine via the activity of glutamine synthetase. This phenomenon may be considered as an anaplerotic reaction to replenish the neurotransmitter pool and for macromolecular synthesis through the TCA cycle in the brain [59]. The differences in metabolic profile between the orthotopic, flank and in vitro settings reflect the plasticity of D425-MED and MED211 MYC amplified medulloblastoma. There are as-yet unknown factors driving de novo glutamine synthesis in orthotopic tumors compared to the emphasis on glutathione production in vitro.

The differences in metabolic data found in D425MED and MED211 among settings reflect that metabolic reprograming is not only the consequence of genetic mutations but also the crosstalk of cancer cells to the microenvironment. Genetics define metabolic pathways to an extent, but the differences in nutrient availability, oxygen levels, pH and interactions with stromal cells in the different environments also regulate metabolic gene expression [52,60].

Metabolic analysis of orthotopic *MYC*-amplified tumors was key for identification of de novo biosynthesis of glutamine, since this was not noted in cells in culture, where glutamine is abundant in the culture media. Based on the abundance of m + 1 glutamate and the absence of m + 6 glutamate in orthotopic tumors, we conclude that the glutaminase II pathway is the main pathway utilizing glutamine in MYC-amplified medulloblastoma. Supporting this conclusion, we identified increased expression of GTK in tumor compared to normal brain. GTK functions to salvage alpha-keto acids, transfer alpha-keto acid/ amino acid carbons between cellular and intracellular compartments, and in the methionine salvage pathway [61]. Uniformly labeled glutamine metabolic analyses demonstrated that the two most abundant glutamine-derived metabolites were glutamate and glutathione in vivo and these were synthesized through the glutamine transaminase K (GTK) enzyme. The glutaminase II pathway is important in normal brain metabolism [61]. However, we believe our report here is the first to describe the glutaminase II pathway as being predominant in *MYC*-amplified medulloblastoma. While we cannot fully exclude the activity of GLS1 in generating an m + 1 amino group that could be incorporated into m + 1 glutamate, the lack of m + 6 glutamate suggests that GLS is not as active in the brain compared to cells in culture and flank tumors and that GTK is the predominant glutaminase enzyme.

The accumulation of glutamine carbons in glutamate rather than in TCA cycle intermediates emphasizes the importance of glutamine and glutamate as key amino acids for *MYC*-driven medulloblastoma and is concordant with our finding that these tumor cells use glucose carbons via the TCA cycle to synthesize glutamate and glutamine (Figure 3E and Figure 4C). Our data suggest there is very little glutamine carbon that contributes to the TCA cycle in *MYC*-amplified medulloblastoma in vivo.

The work presented here also identifies some key metabolic differences between normal cerebellum and cortex and *MYC*-amplified medulloblastoma tumors. Specifically, we identified upregulation of multiple amino acids, such as proline, glycine, histidine and asparagine in medulloblastoma compared to normal brain. We also found upregulation of urea cycle components citrulline and ornithine. The prominence of arginine biosynthesis in the pathway impact analysis (Figure 1C,D) highlights the upregulation of the citrulline–arginine cycle, in both *MYC*-driven medulloblastoma orthotopic xenograft models compared to normal brain. Arginine is used for the synthesis of polyamines, nitric oxide and urea [48,49,62,63]. Polyamines promote oncogenesis by supporting production of nucleic acids and proteins and regulating chromatin and transcription [53,64]. This work also confirmed and extended our prior finding that glutathione was upregulated in *MYC*-amplified medulloblastoma compared to normal brain [40]. Validating our metabolic data, we found that enzymes regulating the TCA and urea cycle are upregulated in medulloblastoma compared to other pediatric brain tumors in the Children’s Brain Tumor Network/KidsFirst Pediatric Brain Tumor Atlas RNAseq dataset. Specifically, we found increased *KYAT*, the mRNA encoding GTK the key step in the glutaminase II pathway. We also found increased arginosuccinate synthetase 1 (ASS1), which reversibly catalyzes the production of arginosuccinate from citrulline and aspartate [65]. This finding is consistent with an independent analysis of a separate dataset that showed high level expression of mRNA for *ASS1*, *ASL* and *ARG2* and low expression of *OTC1* in primary medulloblastoma tumors [49]. We also found increased ornithine decarboxylase 1 (ODC1), which catalyzes the conversion of ornithine to putrescine, the first step in polyamine production. Lastly, we found increased ATP-citrate lyase (ACLY), which cleaves citrate to release acetyl-CoA and produce oxaloacetate [66]. Increased activity of ACLY allows the TCA cycle to run in reverse to potentially produce fumarate to fuel the urea cycle, as shown in Figure 8A.

Targeting these novel vulnerabilities may lead to improved outcomes in *MYC*-amplified medulloblastoma. We previously demonstrated that disrupting glutathione metabolism extended survival of mice bearing *MYC*-driven medulloblastoma orthotopic xenografts, further validating our metabolic findings and showing the value of our metabolic profiling approach [40].The drug difluromethylornithine (DFMO) blocks ODC1 and has had success in high-risk neuroblastoma clinical trials [67,68,69]. There are encouraging preclinical data combining DFMO and the polyamine transporter inhibitor in diffuse intrinsic pontine glioma [70], and this pathway is upregulated in Hedgehog-driven medulloblastoma as well [71].

Multiple drugs are in development in other tumors that target the urea cycle through inhibition of aspartate or ASS1 [72,73], and some of these agents could be applied to medulloblastoma tumors with vulnerable metabolic profiles. Recent publications suggest that clinical metabolic profiling can be performed on pediatric primary tumor samples, suggesting that targeting metabolic profiles could be a new frontier in pediatric cancer and in brain tumors in general [74].

Limitations of our study include a lack of primary human tumor samples for metabolic profiling. We also use only two human cell models of MYC-amplified medulloblastoma. Uniformly labeled metabolomic experiments in MED211 were performed in vitro and in flank tumors. The high degree of concordance between MED211 and D425MED in all analyses suggests reliance on common metabolic pathways in MYC-amplified medulloblastoma. In addition, these limitations are addressed in part by the corroborating data from our laboratory and other groups, showing increased expression of mRNA and proteins related to the synthesis of keymetabolic targets in medulloblastoma patient tumors compared to other pediatric brain tumors [49,54,71]. Our laboratory has already validated the increased reliance on glutathione as a vulnerability by demonstrating the in vivo combinatorial efficacy of treatments that decrease glutathione and chemotherapy, such as carboplatin, that are detoxified by glutathione [40].

## 5. Conclusions

The metabolism of MYC-amplified medulloblastoma cancer cells is different in vitro compared to in vivo. Our study revealed the limitations of metabolic profiling conducted in non-native tumor environments (in cell culture and flank xenografts). We identified multiple metabolites that are altered in orthotopic MYC-amplified medulloblastoma xenografts compared to normal brain and showed in a large clinical dataset that the mRNAs for key enzymes in these pathways are upregulated. Targeting these pathways may represent novel therapeutic approaches for medulloblastoma patients.

## Data Availability

The metabolomics data from this study will be uploaded to the NIH Common Fund’s National Metabolomics Data Repository (NMDR) and will be available from the corresponding author on request.

## Supplementary Materials

The following are available online at https://www.mdpi.com/article/10.3390/cancers14051311/s1, Figure S1: Histology of high MYC amplified medulloblastoma orthotopic D425MED, Figure S2: Orthotopic MYC-amplified medulloblastoma has upregulated TCA intermediates compared to normal brain. Figure S3: In vivo preponderance of glutaminase II pathway in generating glutamine-derived glutamate and glutamine-derived glutathione. Figure S4: Increased expression of KYAT1, the gene encoding GTK, in primary pediatric medulloblastoma compared to other pediatric brain tumor samples, Figure S5: Uncropped Western blots used in this manuscript.

## Abbreviations

α-KG = alpha-ketoglutamate, HE = hematoxylin and eosin, HBP = hexosamine biosynthetic pathway, PCA = principal component analysis, PPP = pentose phosphate pathway, TCA = tricarboxylic acid, GTK = glutamine transaminase K, KGM = alpha-ketoglutaramate.
