# Supplementary material for: Comprehensive Metabolic Profiling of MYC-Amplified Medulloblastoma Tumors Reveals Key Dependencies on Amino Acid, Tricarboxylic Acid and Hexosamine Pathways
Source: Cancers (Basel). 2022 Mar 3;14(5):1311. doi: 10.3390/cancers14051311 (PMC8909278; doi:10.3390/cancers14051311)
Supplement: Supplementary file 1 [file cancers-14-01311-s001.zip › cancers-1596427-Supplementary.pdf]

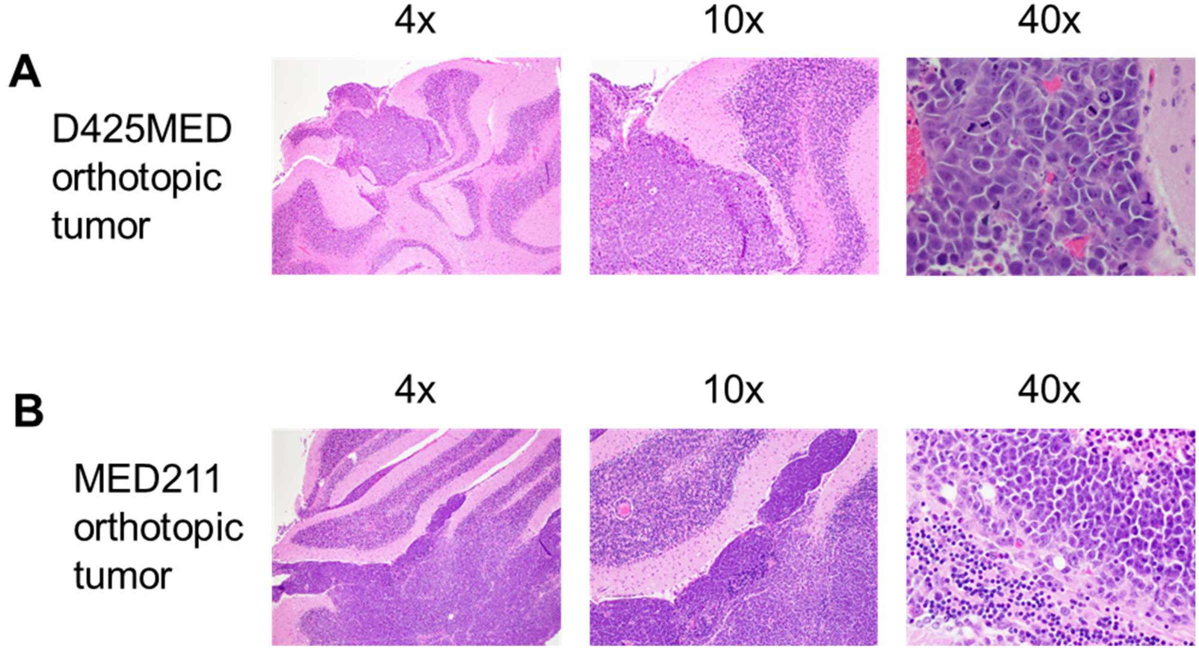

Figure S1. Histology of high MYC amplified medulloblastoma orthotopic D425MED (**A**) and MED211 (**B**) tumors grown in normal mouse cerebellum. High power (40×) shows large cell histology, consistent with Group 3, MYC-amplified medulloblastoma.

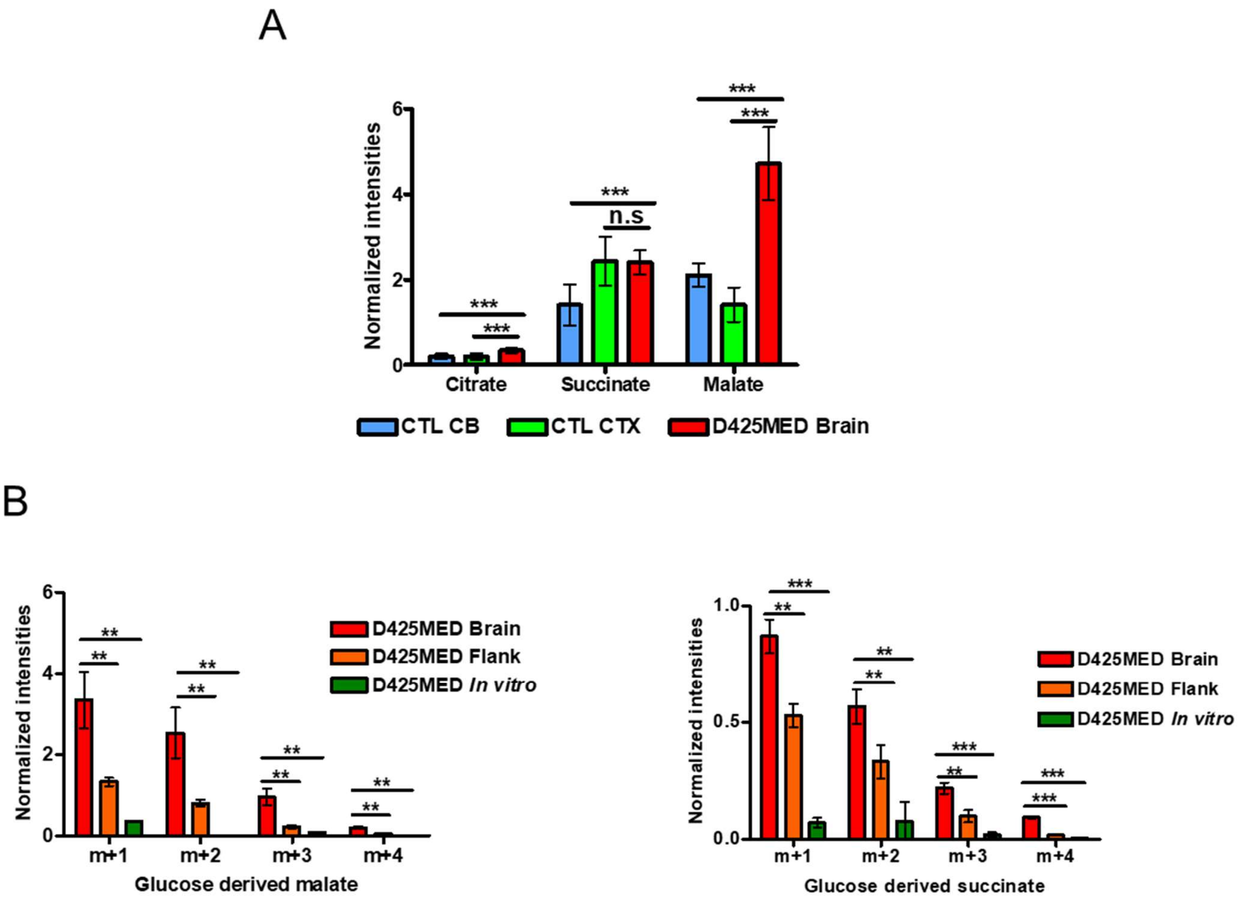

Figure S2. (**A**). Bar graph showing increased intermediate metabolites of TCA cycle (citrate, malate and succinate) in orthotopic D425MED tumors compared to normal brain. (**B**) Bar graphs showing higher intensities of intermediate metabolites of TCA cycle (malate and succinate) found in D425MED orthotopic tumors compared to D425MED flank xenografts and D425MED cells in culture. Abbreviations: normal (control) cerebellum = CTL CB; normal (control) cortex = CTL CTX; D425MED orthotopic tumor = D425MED brain; D425MED flank xenograft tumor = D425MED flank. The bar graph shows the mean intensities with the SD as error bar. Each group has three biological replicate samples. n.s. not significant, ** $p < 0.01$, *** $p < 0.001$, Student *t*-test.

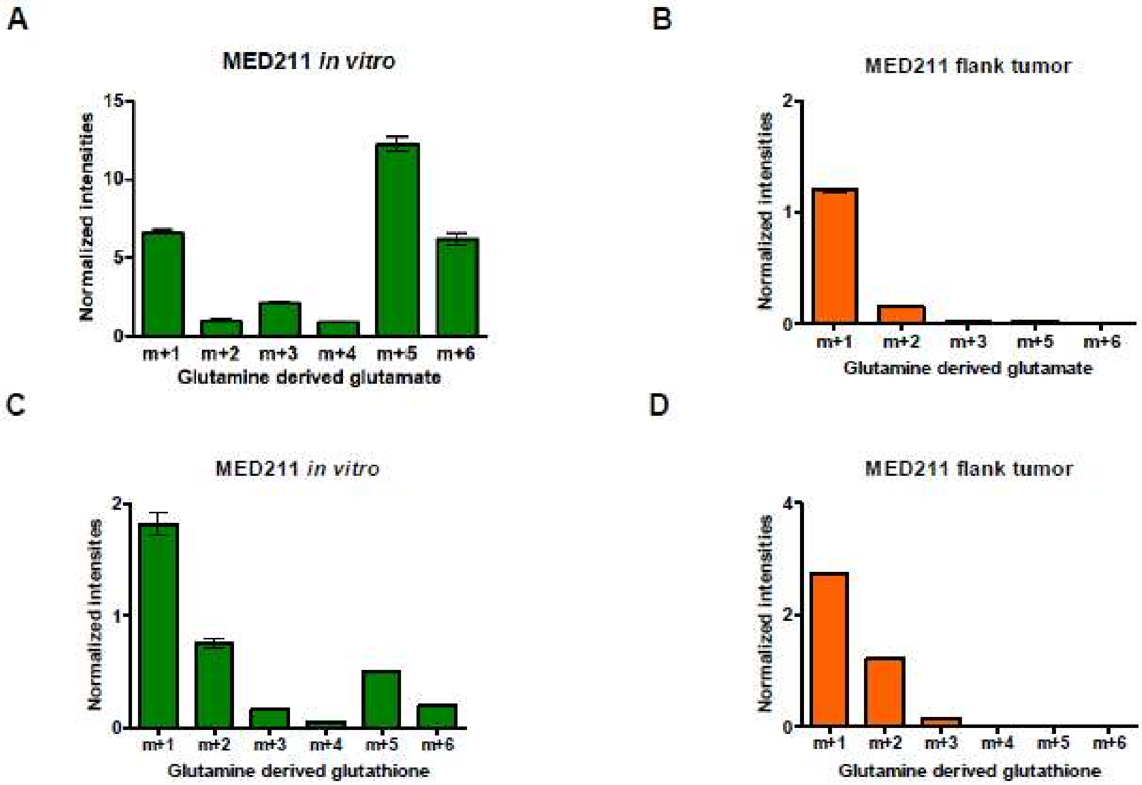

Figure S3. In vivo preponderance of glutaminase II pathway in generating glutamine-derived glutamate and glutamine-derived glutathione. (**A**) Bar graph showing the intensities of glutamine-derived glutamate isotopes found in MED211 cells in culture, showing presence of m + 1 and m + 6. (**B**) Bar graph showing the intensities of glutamine-derived glutamate isotopes found in the MED211 flank tumor, showing predominance of m + 1. (**C**) Bar graph showing the intensities of glutamine-derived glutathione isotopes found in MED211 cells in culture, showing the presence of m + 1 and m + 6, but predominance of m + 1. (**D**) Bar graph showing the intensities of glutamine-derived glutathione isotopes found in the MED211 flank tumor, showing predominance of m + 1 and absence of m + 6.

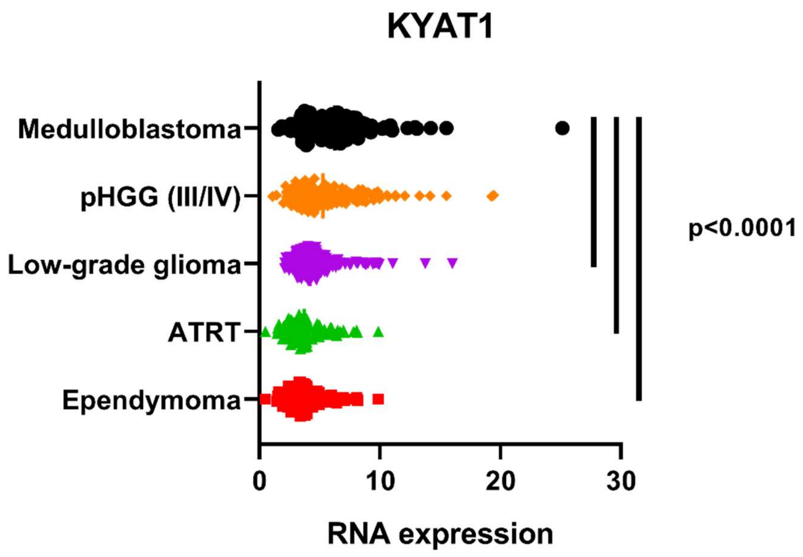

Figure S4. Increased expression of *KYAT1*, the gene encoding GTK in primary pediatric medulloblastoma compared to other pediatric brain tumor samples. Analysis of the Children's Brain Tumor Network Pediatric Brain Tumor Atlas RNAseq dataset (PedsCBioportal) shows upregulation of the mRNA encoding GTK (*KYAT1)* in medulloblastoma compared to other pediatric brain tumor subtypes. *p* values indicated at the right represent results of one way ANOVA with multiple comparisons correction. Each dot represents RNA from a single tumor. Vertical bar in each tumor condition shows mean intensity of normalized RNA expression.

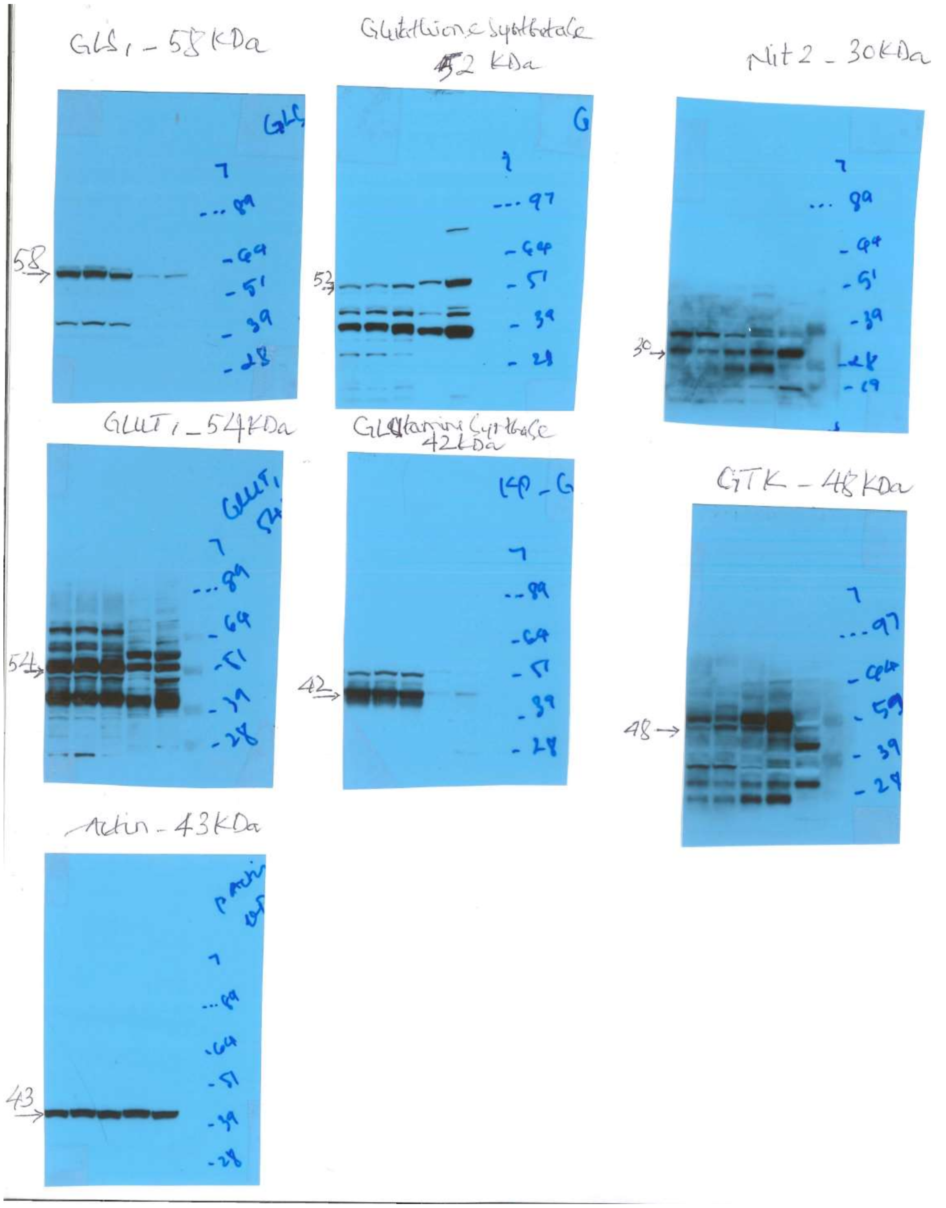

Figure S5. Uncropped Western blots.
